# Giant left atrial thrombus formation in patient with a previous coronary artery bypass grafting

**Published:** 2013-09-25

**Authors:** N Erdil, OM Disli, J Yagmur, S Secici, K Donmez, B Akca, B Battaloglu

**Affiliations:** * Department of Cardiovascular Surgery, Inonu University, Malatya/ Turkey; ** Department of Cardiology, Inonu University, Malatya/Turkey

**Keywords:** Thrombosis, left atrium, coronary artery bypass surgery, reoperation

## Abstract

Free-floating left atrial ball thrombus is a rare condition. We report a giant left atrial ball thrombus which was removed under surgery uneventfully, in a 48-year-old male patient with the presence of sinus rhythm and no valvular disease with previous off-pump coronary artery bypass surgery.

## Introduction

Free-floating left atrial ball thrombus is an extremely rare and serious disorder that usually occurs in the setting of a large, dilated left atrium with stagnant flow, commonly the result of severe rheumatic mitral stenosis and accompanying atrial fibrillation [**1**]. A left atrial free floating ball thrombus is a relatively rare event, especially without a mitral valve disease [**2**]. Although rare, it is important because of its potentially fatal effects. The thrombus can produce sudden circulatory arrest by obstructing the mitral orifice or can cause severe cerebral or peripheral embolic events [**3**]. The following report describes the very unusual case of a patient who underwent an off-pump coronary artery bypass (OPCAB) surgery with a large free-floating left-atrial thrombus. 

### Case Report

**Figure 1:**
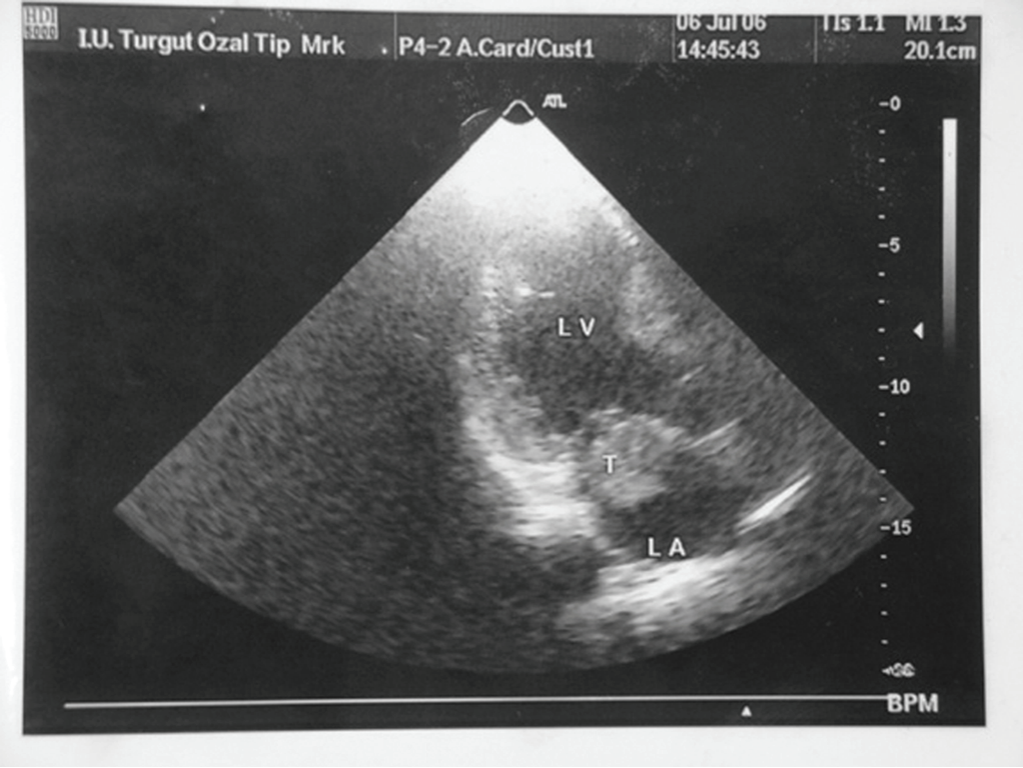
Transesophageal echocardiographic visualization of the thrombus

 A 48 year-old male admitted to our hospital with the complaint of palpitation lasting for two months. One year before, LIMA-LAD and radial artery-right coronary anastomosis had been done with OPCAB surgery. The electrocardiograms and transthoracic echocardiography (TTE) which were done before the coronary artery bypass grafting surgery showed a sinus rhythm and no thrombus in cardiac chambers. During admission, the patient was still in sinus rhythm. He had been receiving aspirin for anticoagulation. On physical examination, the blood pressure was of 110/70 mmHg and the heart rate was regular at 75 beats/min. He had no symptoms or complications such as embolism or syncope. TTE and transesophageal echocardiography (TEE) showed a 3.0x2.8 cm free floating ball thrombus in the left atrium (LA) (**Fig. 1**). TTE showed normal left ventricular dimensions with a moderate reduction in left ventricular ejection fraction (%40) and hypokinesia of the septal and anterior walls. The left atrium was noted to be of normal dimensions. 

**Figure 2:**
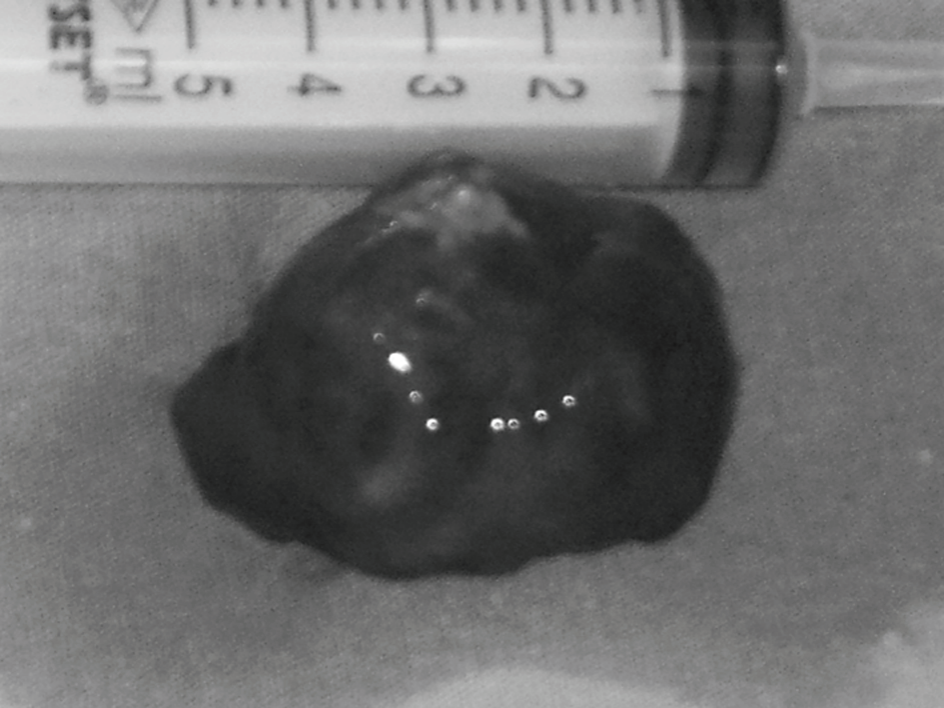
Imaging of the free-floating ball thrombus

 There was no evidence of valvular disease. No coagulation disorder was detected and the hemostatic parameters were within normal ranges. Just prior to the operation, the patient underwent a coronary angiography and the grafts were detected to be patent. The patient subsequently underwent an urgent cardiac operation. The intervention was carried out through a median sternotomy. Cardiopulmonary bypass was established with femoral artery cannulation and bicaval venous drainage. The aorta was cross clamped without damaging the grefts. Cardiac arrest and myocardial protection were obtained with antegrade cold blood cardioplegia. The LA was opened longitudinally and the thrombus was removed. The thrombus was round, smooth, 4 cm in diameter (**Fig. 2**). 

There was no attachment of the thrombus to the atrial wall and no mural thrombus. The mitral valve was within the limits of normality. No stenotic aspect was disclosed, and valvular competence was normal. The left atrium was closed and the cardiopulmonary bypass was discontinued. Postoperatively, he received anticoagulation with warfarin. The postoperative course was uneventful and he was discharged from the hospital on the 7th postoperative day.

## Discussion

 A free-floating ball thrombus is most commonly detected in patients with mitral stenosis, particularly when associated with severe left atrial dilatation, atrial fibrillation, and congestive heart failure [**3**]. It may cause systemic emboli or left ventricular inflow obstruction, often resulting in sudden death. It is rarely detected in the presence of sinus rhythm and, if it is, additional underlying cardiac abnormalities should be sought [**4**]. Its incidence among patients with sinus rhythm is reported to be only of 0.1% [**5**]. The patients with sinus rhythm who had specific cardiac abnormalities, especially significant left ventricular dysfunction, valvular heart disease, and previous episodes of atrial fibrillation are at risk for LA thrombus formation. It is usually diagnosed by transthoracic echocardiography, but the transesophageal approach offers a much better resolution and delineation of the whole mass. Lin et al found that TEE detected LA thrombi with a sensitivity and specificity of 100%, but TTE detected LA thrombi with a sensitivity of 69.2% and a specifitiy of 100% [**6**]. 

 The investigators concluded that TEE is superior to TTE for the detection of LA thrombi. Also, there is another case in literature which TTE examinations did not detect on a left atrial appendage thrombus on 2 separate occasions, but TEE disclosed the thrombus in the presence of the sinus rhythm during CABG. The perioperative use of TEE is considered as category I indication for valve repair, complicated cardiac surgery and for the evaluation of acute haemodynamic instability in the operating room. On the other hand, its use in patients undergoing CABG is considered as category II [**7**]. We do not use TEE for low risk surgery such as CABG by using the OPCAB technique like many other centers. In our case, TEE examination was not performed before or during CABG, so we do not know whether there was a thrombus or not in LA during CABG, or if there was a small thrombus which could not be detected by TTE and enlarged during this period. Recently, a hypercoagulable state after OPCAB surgery has been pointed out by several authors. An increased tendency for hypercoagulable state after OPCAB surgery may be responsible for the thrombus formation or enlargement. Cartier and Robitaille reported thrombotic complications including fatal ones after OPCAB surgery [**8**].

 Another possible mechanism for LA thrombus in our patient is that the patient experienced palpitations for two months and possible paroxysmal AF attacks the patient had, caused a thrombus formation. The patient might also have had an episode of AF causing thrombus formation throughout his previous surgery. Transient paroxysms of AF may result in atrial dysfunction during the arrhythmia and after conversion to sinus rhythm (the phenomenon of atrial stunning), thus predisposing to LA thrombus formation [**9**]. Therefore, LA thrombus may be detected in the presence of the sinus rhythm with or without a clinical recognition of the preceding atrial arrhythmia.

 Significant LV systolic and/or diastolic dysfunction predispose to LA thrombus formation via their secondary effect on LA hemodynamic [**10**]. As LV dysfunction is usually associated with an abnormal mitral flow pattern and reduced filling in the left atrial appendix (LAA), the patient is at risk of stasis of blood in the LAA [**11**]. Our patient had moderate LV dysfunction which might have predisposed LA thrombus formation. 

 In conclusion, we report a giant left atrial ball thrombus which was removed under surgery, uneventfully, in a patient with the presence of sinus rhythm and no valvular disease with previous CABG.
